# Location, synthesis and function of glycolipids and polyglycerolphosphate lipoteichoic acid in Gram-positive bacteria of the phylum *Firmicutes*

**DOI:** 10.1111/j.1574-6968.2011.02260.x

**Published:** 2011-03-25

**Authors:** Nathalie T Reichmann, Angelika Gründling

**Affiliations:** Section of Microbiology, Imperial College LondonSouth Kensington Campus, London, UK

**Keywords:** LTA, LtaS, cell wall, Gram-positive periplasm, protein localization

## Abstract

Lipoteichoic acid (LTA) is a zwitterionic polymer found in the cell wall of many Gram-positive bacteria. A widespread and one of the best-studied forms of LTA consists of a polyglycerolphosphate (PGP) chain that is tethered to the membrane via a glycolipid anchor. In this review, we will summarize our current understanding of the enzymes involved in glycolipid and PGP backbone synthesis in a variety of different Gram-positive bacteria. The recent identification of key LTA synthesis proteins allowed the construction and analysis of mutant strains with defined defects in glycolipid or backbone synthesis. Using these strains, new information on the functions of LTA for bacterial growth, physiology and during developmental processes was gained and will be discussed. Furthermore, we will reintroduce the idea that LTA remains in close proximity to the bacterial membrane for its function during bacterial growth rather than as a surface-exposed structure.

## Introduction

The typical cell wall of low G+C Gram-positive bacteria, which include the well-studied model organism *Bacillus subtilis* and many important pathogens such as *Staphylococcus aureus, Enterococcus faecalis, Streptococcus agalactiae, Streptococcus pyogenes* and *Listeria monocytogenes*, is composed of proteins, peptidoglycan and teichoic acids (TAs). TAs can be further grouped into wall teichoic acid (WTA) and lipoteichoic acid (LTA), the latter of which is the focus of this review. The chemical structure of these cell wall components and the enzymes involved in peptidoglycan and WTA synthesis have been known for some time. However, our understanding of the synthesis and function of LTA has recently come into focus with the identification of key enzymes involved in its production. This has allowed the construction of bacterial strains that lack LTA, which in turn could be used to study the function of this polymer. These recent studies corroborate earlier suggestions of important roles for LTA in bacterial growth, physiology and during developmental processes, which will be discussed.

**Fig. 1:**
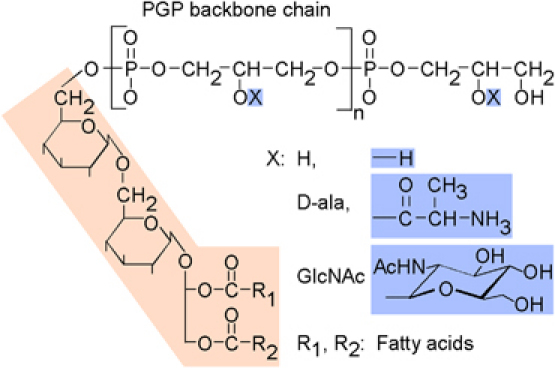
Chemical structure of PGP-type LTA. Schematic representation of *Staphylococcus aureus* and *Bacillus subtilis* LTA, which consists of a PGP chain that is linked to a diglucosyldiacylglycerol lipid anchor (boxed in orange). The hydroxyl groups at position C2 of the GroP subunits are substituted with d-alanine residues in both organisms and additional *N*-acetylglucosamine modifications are found in *B. subtilis* (shown in blue).

Both WTA and LTA are zwitterionic cell wall polymers; however, their synthesis proceeds through separate pathways. WTAs are usually composed of ribitol phosphate, glycerolphosphate (GroP) or more complex sugar-containing polymers that are polymerized on an undecaprenyl-phosphate precursor within the cytoplasm and transported across the membrane before being covalently linked to the peptidoglycan (Xia & Peschel, 2008). LTA has a simpler structure that typically consists of a polyglycerolphosphate (PGP) chain that is linked via a glycolipid anchor to the bacterial membrane (Fischer, 1990) (Fig. 1). The GroP backbone chain of both WTA and LTA is modified with alanine residues and in many bacteria with additional glycosyl groups (Fig. 1). The PGP chain is polymerized on the outside of the cell and the GroP subunits are derived from the head group of the membrane lipid phosphatidylglycerol (PG) and most likely added to the distal end of the growing chain (Taron *et al.*, 1983; Koch *et al.*, 1984).

Here, we will summarize our current understanding of glycolipid and PGP-LTA backbone synthesis, and highlight enzymes that are involved in these synthesis processes in several well-studied Gram-positive organisms. We will discuss the function of glycolipids and LTA in terms of their importance for cell growth, cell shape and virulence, which emerged based on phenotypes observed in defined mutants. LTA backbone chain modifications will not be discussed further as the incorporation and function of d-alanine modification has only recently been reviewed (Neuhaus & Baddiley, 2003) and the process and function of glycosyl modifications is largely unknown. In addition, we will emphasize and reintroduce a theory for the localization of LTA in close proximity to the bacterial membrane (Fischer, 1994), which is important for bacterial growth and cell division, rather than LTA as a surface-exposed structure.

## Glycolipid production: the LTA anchor

LTA production begins in the cytoplasm of the cell with the synthesis of the glycolipid anchor, which tethers the PGP chain to the cell membrane. In most cases, the LTA anchor is a glycolipid consisting of a disaccharide linked to diacylglycerol (DAG) (Fischer, 1988) that is produced by either a single processive glycosyltransferase or two separate enzymes. No uniform nomenclature is used to describe these glycosyltransferases that use nucleotide-activated sugars for the production of glycolipids, and in most cases, these enzymes are named based on observed phenotypes in deletion strains.

**Fig. 2:**
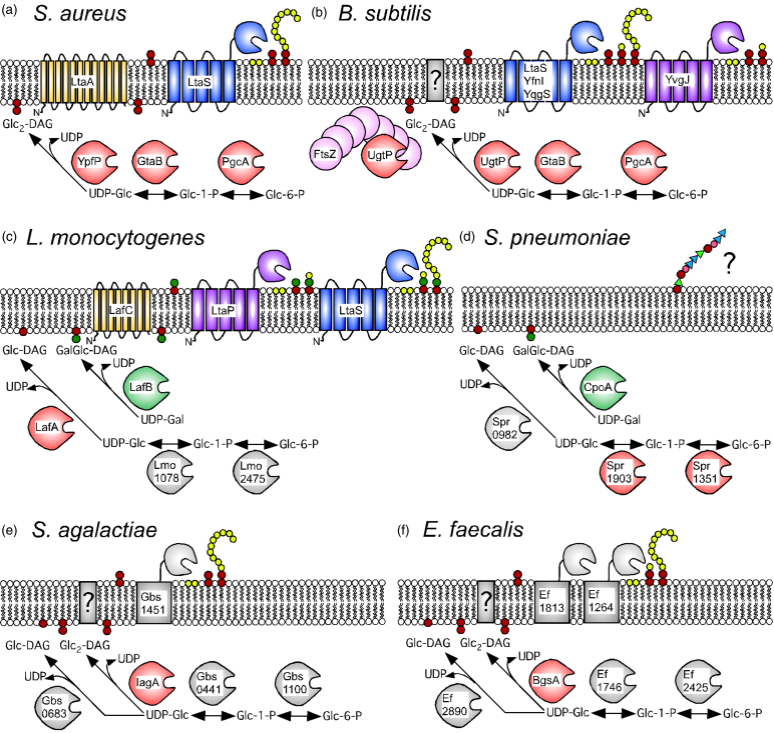
Enzymes required for glycolipid and LTA backbone synthesis in different Gram-positive bacteria. Schematic representation of enzymes involved in glycolipid precursor, glycolipid and/or LTA backbone synthesis in (a) *Staphylococcus aureus*, (b) *Bacillus subtilis*, (c) *Listeria monocytogenes*, (d) *Streptococcus pneumoniae*, (e) *Streptococcus agalactiae* and (f) *Enterococcus faecalis*. Enzymes with experimental evidence for their activity are shown in color and their function is described in the text, while enzymes identified by blast searches based on homology are shown in gray. Predicted but still unidentified proteins are indicated with question marks (?). Glycolipid synthesis precursors are UDP nucleotide-activated sugars that are produced in the cytoplasm of the cell. These precursors are used by glycosyltransferases to synthesize the LTA glycolipid anchor by the transfer of glucose (red circles) or galactose (green circle) moieties onto the membrane lipid DAG. After transport of the glycolipid from the inside to the outside of the membrane, GroP subunits (yellow circles) derived from the membrane lipid phosphatidylglycerol are added to the anchor and the chain is extended by the repeated addition of GroP subunits at the distal end. This leads to the formation of the PGP-LTA backbone chain, which is schematically depicted by a string of yellow circles. In contrast, the non-PGP-type LTA in *S. pneumoniae* (d) is depicted by different symbols. Proteins are either named according to the information available in the literature or based on *L. monocytogenes* EGD-e, *S. pneumoniae* R6, *S. agalactiae* NEM316, and *E. faecalis* V583 gene numbers. Abbreviations used are as follows: Glc-6-P, glucose-6-phosphate; Glc-1-P, glucose-1-phosphate; Glc_2_-DAG, diglucosyldiacylglcerol; GalGlc-DAG, galactosylglucosyldiacylglycerol.

The LTA anchor in *S. aureus* and *B. subtilis* is composed of a glucosyl (β1-6) glucosyl (β1-3) DAG (Glc_2_-DAG) that is generated by the transfer of two glucose moieties from uridine diphosphate glucose (UDP-Glc) to DAG (Jorasch *et al.*, 1998, 2000; Kiriukhin *et al.*, 2001). As shown in Fig. 2a and b, UDP-Glc itself is produced by conversion of glucose-6-phosphate to glucose-1-phosphate by the α-phosphoglucomutase PgcA (also named Pgm), followed by activation by the UTP:α-glucose-1-phosphate uridyltransferase GtaB (sometimes named GalU) (Pooley *et al.*, 1987; Lu & Kleckner, 1994; Lazarevic *et al.*, 2005; Gründling & Schneewind, 2007a). A processive glycosyltransferase (YpfP in *S. aureus* and UgtP in *B. subtilis*) transfers two glucose moieties from UDP-Glc to DAG, leading to the formation of the glycolipid Glc_2_-DAG (Jorasch *et al.*, 1998; Kiriukhin *et al.*, 2001). In the absence of *pgcA, gtaB* or *ypfP*, no glycolipids are produced, but the LTA backbone is still synthesized. In these mutants, LTA is believed to be linked directly to DAG, a simpler lipid anchor also naturally used by some bacteria (Iwasaki *et al.*, 1986; Lazarevic *et al.*, 2005; Gründling & Schneewind, 2007a).

*Listeria monocytogenes* uses galactosyl (α1-2) glucosyl (α1-3) DAG (GalGlc-DAG) as the glycolipid anchor of LTA (Uchikawa *et al.*, 1986) and therefore requires two distinct glycosyltransferases, LafA (LTA anchor formation protein A; Lmo2555) and LafB (LTA anchor formation protein B; Lmo2554), to produce monoglucosyldiacylglycerol (Glc-DAG) and GalGlc-DAG, respectively (Webb *et al.*, 2009). Neither of the enzymes involved in UDP-Glc or UDP-galactose (UDP-Gal) production have been characterized in any detail, but homologues of *S. aureus* and *B. subtilis* PgcA and GtaB can be identified using blast searches (Fig. 2c). An additional phosphatidyl lipid can be linked to the glucose moiety of the glycolipid in *L. monocytogenes*, although the enzyme required for its formation is unknown (Uchikawa *et al.*, 1986). Recently, it was found that at a higher temperature (37 °C vs. 30 °C), the fraction of LTA that contains this modified glycolipid anchor is increased (Dehus *et al.*, 2011). It was further shown that LTA with this additional lipid modification less efficiently activates the human complement system through the lectin pathway, and it was speculated that this modification could help bacteria survive during an infection (Dehus *et al.*, 2011).

LTA anchor formation has also been investigated in *S. agalactiae* and *E. faecalis*, where the α1-2-linked Glc_2_-DAG glycolipid is produced by a two-enzyme system similar to *L. monocytogenes* (Doran *et al.*, 2005; Theilacker *et al.*, 2009). The *S. agalactiae* glycosyltransferase IagA (invasion-associated gene A) and the *E. faecalis* glycosyltransferase BgsA (biofilm-associated glycolipid synthesis gene A) are responsible for the addition of the second glucose moiety and strains deleted for the respective genes accumulate Glc-DAG in the membrane (Doran *et al.*, 2005; Theilacker *et al.*, 2009). Both IagA and BgsA are homologous to the *L. monocytogenes* LafB and are encoded in an operon with *gbs0683* and *ef2890*, respectively, that displays similarity to *lafA*, presumably encoding the glycosyltransferase responsible for the production of Glc-DAG (Fig. 2e and f).

Following synthesis in the cytoplasm, a fraction of the glycolipids are transported to the outer leaflet of the membrane, where they are used as the LTA anchor. *Staphylococcus aureus* LtaA, a member of a major facilitator superfamily clan that is encoded in an operon with *ypfP*, has been suggested to be involved in this process (Gründling & Schneewind, 2007a). This was based on the observation that in the absence of LtaA, the majority of LTA is linked directly to DAG despite the presence of wild-type levels of Glc_2_-DAG (Gründling & Schneewind, 2007a). In *L. monocytogenes*, an additional membrane protein, LafC, is encoded in an operon with *lafA* and *lafB*. However, LafC does not show any homology to the *S. aureus* LtaA protein and while slight changes in glycolipid and the LTA structure could be detected in its absence, the observed alterations were not necessarily consistent with a function in glycolipid transport (Webb *et al.*, 2009). Furthermore, no apparent homologues to LafC or LtaA are present in *B. subtilis*. It is commonly accepted that a protein component is involved in the membrane transfer of glycolipids. However, it remains to be determined whether indeed, as it appears, a variety of nonhomologous membrane proteins can perform this reaction.

## PGP production: the LTA backbone

Once the glycolipid reaches the outer leaflet of the membrane, in *S. aureus*, a single LTA synthase enzyme (LtaS) polymerizes the GroP to generate the PGP chain (Gründling & Schneewind, 2007b) (Fig. 2a). *Staphylococcus aureus* LtaS and its homologues in other Gram-positive bacteria are predicted membrane proteins with a large extracellular enzymatic domain, which is consistent with LTA synthesis occurring on the outer side of the membrane (Gründling & Schneewind, 2007b; Lu *et al.*, 2009). Based on pulse-chase experiments, it has been suggested that the GroP subunits are derived from the membrane lipid PG (Koch *et al.*, 1984), and recently, it has been shown that the purified enzymatic domain of LtaS can cleave the GroP head group of fluorescently labeled PG to form DAG, providing further evidence for this notion (Karatsa-Dodgson *et al.*, 2010). In contrast, two LtaS-type enzymes produce the PGP backbone in *L. monocytogenes*. The LTA primase LtaP (Lmo0644) adds the first GroP subunit to the GalGlc-DAG lipid anchor and LtaS (Lmo0927) polymerizes the LTA backbone chain (Fig. 2c) (Webb *et al.*, 2009). In the absence of the primase, LTA is still produced, but the deletion of *ltaS* abolishes LTA synthesis.

The situation is ever more complex in *B. subtilis*, which encodes four LtaS-type enzymes: LtaS (originally YflE), YqgS, YvgJ and YfnI (Gründling & Schneewind, 2007b; Schirner *et al.*, 2009). All four proteins can hydrolyze fluorescently labeled PG *in vitro* and the expression of LtaS, YqgS or YfnI as the sole LTA synthesis enzyme is sufficient for PGP-LTA production (Wörmann *et al.*, 2011) (Fig. 2b). Expression of YvgJ leads to the accumulation of GroP-Glc_2_-DAG, indicating that it functions as an LTA primase, similar to *L. monocytogenes* LtaP (Wörmann *et al.*, 2011). LtaS appears to be the most important enzyme of the four proteins and the other homologues are thought to play overlapping roles in response to changing environmental conditions. YfnI expression is controlled by the stress σ-factor σM, while YqgS function is important during the sporulation process (Thackray & Moir, 2003; Jervis *et al.*, 2007; Eiamphungporn & Helmann, 2008; Schirner *et al.*, 2009).

Two different models for LTA synthesis have been proposed that differ in the enzymatic activity that is required for the linkage of the PGP chain to the glycolipid anchor and these have been reviewed recently (Rahman *et al.*, 2009b; Sutcliffe, 2011). In one model, it was proposed that an ‘LTA transferase’ moves fully synthesized PGP polymers from a DAG lipid anchor onto the glycolipid anchor. The second model suggests that an LTA primase adds the first GroP subunit to the glycolipid to form a GroP-glycolipid intermediate and subsequently an LTA synthase extends the GroP chain to produce the LTA backbone. The discovery of enzymes with LTA primase activity in *L. monocytogenes* and *B. subtilis* strongly favors the second model (Webb *et al.*, 2009; Sutcliffe, 2011; Wörmann *et al.*, 2011). However, in addition to this two-enzyme system, a slightly altered version of the latter model, in which a single enzyme can directly start and extend the GroP chain on a glycolipid anchor, is also used in some bacteria such as *S. aureus*. The reason why some bacteria use one enzyme while others use multiple enzymes to synthesize the LTA backbone is currently not understood, but it is thought that multiple enzymes allow bacteria to fine-tune LTA synthesis in changing environments and during developmental processes such as sporulation in *B. subtilis*.

It is interesting to note that recent literature has highlighted the presence of a PGP-type LTA in various *Actinobacteria* (Rahman *et al.*, 2009a,c). However, these bacteria appear to lack LtaS homologues and, therefore, it has been suggested that the polymer is synthesized by an alternative pathway (Rahman *et al.*, 2009c).

## *Streptococcus pneumoniae* LTA: an exception to the PGP-type LTA

In contrast to the *Firmicutes* discussed above, other members of this phylum including *Lactococcus garvieae, Clostridium innocuum* and *S. pneumoniae* are known to produce LTA with more complex structures (Fischer, 1990; Greenberg *et al.*, 1996). Recent progress has been made regarding LTA synthesis in the extensively studied Gram-positive pathogen *S. pneumoniae*, where LTA is known to consist of *N*-acetylgalactosamines, ribitol phosphate, glucose and acetamido-4-amino-2,4,6-trideoxy-d-galactose (AATGal) repeating units (Behr *et al.*, 1992; Seo *et al.*, 2008). Consistent with the lack of a PGP-type LTA, no LtaS-type enzyme is present in *S. pneumoniae*. Despite this, it is still assumed that the LTA backbone is linked to a membrane glycolipid. Initially, it was proposed that this lipid anchor was Glc-AATGal-Glc-DAG (Behr *et al.*, 1992), but this glycolipid has never been detected in the membrane. More recently, Seo *et al.* (2008) proposed that the anchor is Glc-DAG, which constitutes a minor fraction of the membrane lipids in *S. pneumoniae*, while GalGlc-DAG is the predominant glycolipid (Brundish *et al.*, 1965). This lipid is likely synthesized in the same manner as in *L. monocytogenes*, requiring the production of UDP-Glc by the phosphoglucomutase Pgm (Spr1351) and uridyltransferase GalU (Spr1903) (Mollerach *et al.*, 1998; Hardy *et al.*, 2001) (Fig. 2d). For GalGlc-DAG production, the transfer of the second sugar moiety using UDP-Gal as the substrate is presumably performed by the glycosyltransferase CpoA, a LafB homologue (Edman *et al.*, 2003), which is encoded in an operon with a second glycosyltransferase (Spr0982) that shows homology to LafA and presumably synthesizes Glc-DAG (Fig. 2d).

While the key enzymes in PGP-LTA synthesis have now been characterized, many more enzymes remain to be identified to decipher the LTA synthesis pathway in *S. pneumoniae*. Because the chain structures of LTA and WTA in *S. pneumoniae* are identical, it is possible that these polymers are, as suggested recently, synthesized by overlapping pathways and deciphering one may also shed light on the other (Seo *et al.*, 2008; Rahman *et al.*, 2009b).

## Phenotypes of mutants defective in glycolipid synthesis

Bacteria with deletions in genes required for glycolipid synthesis are viable, but display diverse phenotypes. *Staphylococcus aureus*Δ*ypfP* cells, which lack the glycolipid anchor and produce LTA bound to DAG, are enlarged, misshaped cocci (Kiriukhin *et al.*, 2001), while *B. subtilis*Δ*ugtP* mutants are shorter (Price *et al.*, 1997; Weart *et al.*, 2007; Salzberg & Helmann, 2008), exhibit a reduction in swarming motility and increased susceptibility to cationic antimicrobial peptides (Salzberg & Helmann, 2008). Depending on the strain background, an *S. aureus ypfP* mutant shows either a slight increase in PGP-LTA production and release into the supernatant or a drastic reduction in LTA production to 10% of the parental strain (Kiriukhin *et al.*, 2001; Fedtke *et al.*, 2007; Gründling & Schneewind, 2007a). The reason for these opposing phenotypes is currently not understood. The *ypfP* mutant strain with reduced LTA amounts shows a defect in biofilm formation (Fedtke *et al.*, 2007) and has a reduced ability to penetrate the blood–brain barrier (Sheen *et al.*, 2010); the latter phenotype was also observed for a group B *Streptococcus iagA* mutant (Doran *et al.*, 2005). The *E. faecalis bgsA* mutant was named because of its defect in biofilm formation and is also defective in adhesion to Caco-2 cells (Theilacker *et al.*, 2009). Subsequently, it was shown that Glc_2_-DAG, but not LTA, was able to inhibit the binding of *E. faecalis* to Caco-2 cells (Sava *et al.*, 2009). This led the authors to propose a model in which LTA released into the supernatant binds to glucosaminoglycans on the eukaryotic membrane via the glycolipid anchor. The PGP chain, which is now tethered to the host cell, is then capable of rebinding to the bacterial cell wall, allowing for enhanced adhesion.

While the observed defects and phenotypes between different Gram-positive glycolipid mutants may vary, some general conclusions can be made. LTA backbone synthesis still proceeds in the absence of glycolipids, indicating that LtaS-type enzymes can initiate LTA synthesis on a different lipid. Mutants show some morphological defects, often a reduced ability to form biofilms, reduced ability to adhere to eukaryotic host cells and cross host cell barriers, which also leads to reduced virulence.

## Morphological defects in bacterial strains lacking PGP-LTA

LtaS-type enzymes need to be inactivated to completely abolish LTA synthesis. So far, strains lacking LTA have been constructed in *S. aureus, B. subtilis* and *L. monocytogenes* (Gründling & Schneewind, 2007b; Schirner *et al.*, 2009; Webb *et al.*, 2009), and very recently, the construction of an LTA-deficient *Lactobacillus acidophilus* strain has been described (Mohamadzadeh *et al.*, 2011). With the exception of *L. acidophilus*, these bacteria display growth and severe morphological defects. However, two LtaS homologues appear to be encoded in the *L. acidophilus* genome and only one of them was inactivated. It will be interesting to further investigate in future studies the contribution of both proteins to LTA synthesis and growth in this bacterium.

Upon deletion of the single *ltaS* gene in *S. aureus*, bacteria increase in size, display aberrant placement of division septa and can only grow in the presence of high sucrose or NaCl concentrations, conditions that presumably provide osmoprotection (Gründling & Schneewind, 2007b; Oku *et al.*, 2009). An *L. monocytogenes ltaS* mutant lacking the PGP polymer shows a temperature-sensitive growth phenotype and forms long filaments that eventually lyse at 37 °C, the nonpermissive growth temperature (Webb *et al.*, 2009). A similar filamentation phenotype was observed in a *B. subtilis ltaS* mutant, where it was further observed that assembly of the FtsZ ring, one of the key initial steps in the cell division process, does not proceed properly (Schirner *et al.*, 2009). However, it should be noted that even in the absence of LtaS, PGP-LTA is still produced in this mutant. Only the combined absence of the three LTA synthases LtaS, YqgS and YfnI leads to a complete lack of the polymer (Wörmann *et al.*, 2011). A quadruple mutant, which is unable to synthesize LTA, shows more severe morphological defects and bacteria form filaments that spiral along their long axes (Schirner *et al.*, 2009). Lastly, it was found that an *ltaS*/*yqgS* double mutant cannot sporulate, highlighting the importance of LTA during this developmental process (Schirner *et al.*, 2009).

Microscopic analysis of bacteria lacking LTA implicates a function for this polymer in the cell division process. This is especially obvious in rod-shape bacteria, where the lack of LTA leads to a filamentation phenotype without drastically affecting the diameter of the cell. Our current understanding of the molecular mechanism that links cell division and LTA synthesis is limited, but will be discussed below.

## Localization of LTA synthesis proteins and their link with the cell division machinery

The protein localization pattern of three *B. subtilis* LTA synthesis enzymes, LtaS, YqgS and UgtP, was investigated and all three proteins were detected at septa, suggesting that glycolipid and LTA synthesis occurs at the site of cell division (Nishibori *et al.*, 2005; Weart *et al.*, 2007; Schirner *et al.*, 2009). Interestingly, the localization pattern of UgtP correlated specifically with the location of the invaginating septum, reminiscent of the localization of FtsZ (Nishibori *et al.*, 2005). Furthermore, Weart *et al.* (2007) found that purified UgtP protein could inhibit FtsZ polymerization in an *in vitro* assay, suggesting a direct physical interaction between these proteins. UgtP localization to the septum was dependent on the presence of UDP-Glc and was abolished in PgcA- and GtaB-deficient strains. It was suggested that *B. subtilis* uses the intracellular UDP-Glc concentration to sense the metabolic state of the cell and fine-tune FtsZ ring assembly, and hence the shorter cell size of *B. subtilis ugtP, pgcA* or *gtaB* mutants is due to the absence of UgtP-dependent inhibition of FtsZ polymerization (Weart *et al.*, 2007). A direct interaction between FtsZ and UgtP in living cells remains to be determined and it is still an outstanding question whether other Gram-positive bacteria use glycosyltransferases to regulate cell division by influencing FtsZ function. While such a mechanism can be used to fine-tune cell division, the block/retardation in FtsZ assembly in the complete absence of LTA must be caused by a different mechanism. How the absence of LTA on the outside of the cell influences FtsZ assembly on the inside of the cell awaits further investigation.

## Location of LTA in the Gram-positive periplasm and its release

**Fig. 3:**
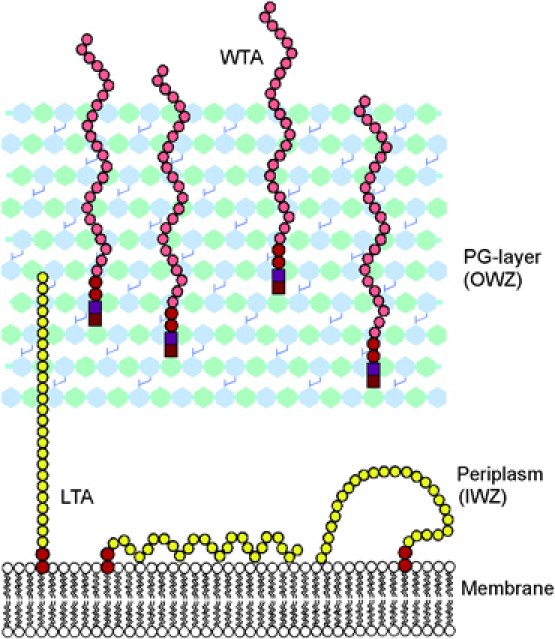
Cell wall of Gram-positive bacteria. This representation of the Gram-positive cell wall is based on cryo-electron microscopy images, in which two clearly distinct cell wall zones were observed outside the membrane: a low-density IWZ, the Gram-positive periplasm, and a high-density OWZ, composed of the peptidoglycan-layer (PG-layer) (Matias & Beveridge, 2005, 2006, 2008). Besides the PG-layer, other components of the Gram-positive cell wall include the peptidoglycan-linked WTA and the membrane-linked LTA polymers, indicated in this figure. LTA, predicted to be a major component of the IWZ, is closely associated with the bacterial membrane during its production and may also remain closely associated with the lipid bilayer once fully synthesized. Also, fully extended membrane-linked LTA may not be able to completely penetrate the peptidoglycan layer and only reach the bacterial surface once released from the membrane.

More than 20 years ago, the existence of a periplasmic space as part of the Gram-positive cell wall was proposed (Takade *et al.*, 1988; Umeda *et al.*, 1992). Recently, this idea was reintroduced by Beveridge and colleagues based on cryo-transmission electron microscopy images of unstained, ultrarapid frozen and hydrated sections of *B. subtilis* and *S. aureus* cells (Matias & Beveridge, 2005, 2006). Two clearly distinct cell wall zones were observed outside the membrane: a low-density inner wall zone (IWZ), which is thought to be the Gram-positive periplasm, and a high-density outer wall zone (OWZ), which consists of the cross-linked peptidoglycan layer (Fig. 3). Based on these images, the thickness of the *B. subtilis* cell wall was estimated to be 55 nm (22.3±4.8 IWZ and 33.3±4.7 OWZ) and that of the *S. aureus* cell wall to be 36 nm (15.8±2.5 IWZ and 19±4.3 OWZ) (Matias & Beveridge, 2005, 2006). Labeling experiments with positively charged gold nanoparticles showed that these particles accumulate uniformly across the IWZ and OWZ, suggesting that the periplasmic space is filled with negatively charged components (Matias & Beveridge, 2008). After removing the OWZ layer with cell wall hydrolytic enzymes, a diffuse layer of approximately 30 nm extended from the outer leaflet of the membrane in *B. subtilis*. This layer was not significantly removed by protease treatment, providing evidence for components of a nonproteinaceous nature, and the fact that it could be labeled with an LTA-specific monoclonal antibody indicated that LTA might be a major component of the Gram-positive periplasm (Matias & Beveridge, 2008). Previous electron microscopy studies and immunoperoxidase labeling experiments using *S. aureus* protoplasts revealed LTA-specific labeling of the bacterial membrane (Aasjord & Grov, 1980). However, surface-exposed LTA could only be detected in whole cells of *S. aureus* strain Cowan I, but not in *S. aureus* strain Wood 46, providing evidence that LTA is not necessarily a cell surface-exposed molecule (Aasjord & Grov, 1980). Indeed, the 30 nm size of the diffuse layer in *B. subtilis* and the estimate that an extended LTA chain of 25 repeating units, as for instance present in *S. aureus*, is <20 nm in length (Labischinski *et al.*, 1991; Fischer, 1994) does not necessarily agree with the commonly depicted schematic representation of the Gram-positive cell wall, in which the membrane-linked LTA chain extends through the cell wall to the surface. In addition, the extension of the PGP chain by the membrane protein LtaS, which uses PG as a substrate and presumably occurs at the distal end (Taron *et al.*, 1983), necessitates that the polymer remains closely associated with the bacterial membrane, at least during its synthesis. The introduction of d-alanines, which can also occur on fully synthesized LTA, suggests that LTA chains remain in contact with the cell membrane even after their synthesis (Fig. 3). Therefore, it seems reasonable to assume that the physiological function of LTA in bacterial growth and cell division might predominantly take place in close proximity to the cell membrane and not at the bacterial cell surface. However, the transfer of d-alanines from LTA to WTA indicates an apparent flexibility, allowing the chain to also extend towards the peptidoglycan layer (Haas *et al.*, 1984) (Fig. 3).

Despite this, it is well documented that LTA is released from the bacterial cell and is frequently found in the culture supernatant and possibly rebinds to the bacterial cell surface (see review; Fischer, 1988). The mechanism by which LTA is released is not understood, but it is thought that it does not depend on actual cell lysis. In all likelihood, this fraction of LTA is not required for bacterial growth and cell division, but may explain how the polymer can play a role in processes such as adhesion and biofilm formation as suggested by others (Sava *et al.*, 2009).

## Concluding remarks

An original suggestion for the function of TAs 40 years ago was an involvement in the retention of ions in the cell wall and controlling ion access to the inner region of the wall (Archibald *et al.*, 1961). Recently, it was shown that a *B. subtilis ltaS* mutant can grow at a low Mg^2+^ concentration, but becomes very sensitive to Mn^2+^ ions, corroborating this early suggestion (Schirner *et al.*, 2009). Recently, the mechanism of ion binding to WTA, which is still linked to peptidoglycan, was reinvestigated using solid-state nuclear magnetic resonance (Kern *et al.*, 2010). Based on these experiments, a model was proposed in which the metal ion binds cooperatively between a peptidoglycan peptide strand and the phosphate group of WTA (Kern *et al.*, 2010). In analogy, one might expect a complex metal-binding mechanism between LTA and the negatively charged phospholipids in the bacterial membrane. While both WTA and LTA may influence the metal-binding ability of the bacterial cell wall, the construction and analysis of defined WTA- and LTA-deficient mutants showed that these polymers have clearly distinct functions in the cell (Schirner *et al.*, 2009). Furthermore, as highlighted in this review, LTA-deficient cells have morphological defects that are consistent with a function of this polymer in the cell division process. For this function, LTA should not be seen as an actual cell surface structure, but more as a polymer in a unique location in the cell wall, in close proximity to the bacterial membrane.

## Statement

Re-use of this article is permitted in accordance with the Term and Conditions set out at http://wileyonlinelibrary.com/onlineopen#OnlineOpen-Terms
